# Estimation of Botanical Composition in Mixed Clover–Grass Fields Using Machine Learning-Based Image Analysis

**DOI:** 10.3389/fpls.2021.622429

**Published:** 2021-02-11

**Authors:** Sashuang Sun, Ning Liang, Zhiyu Zuo, David Parsons, Julien Morel, Jiang Shi, Zhao Wang, Letan Luo, Lin Zhao, Hui Fang, Yong He, Zhenjiang Zhou

**Affiliations:** ^1^College of Biosystems Engineering and Food Science, Zhejiang University, Hangzhou, China; ^2^School of Agricultural Engineering, Jiangsu University, Zhenjiang, China; ^3^Department of Agricultural Research for Northern Sweden, Swedish University of Agricultural Sciences, Umeå, Sweden; ^4^Hangzhou Academy of Agricultural Sciences, Hangzhou, China

**Keywords:** crop species classification, forage crop, transfer learning, DeepLab V3+, back propagation neural network

## Abstract

This study aims to provide an effective image analysis method for clover detection and botanical composition (BC) estimation in clover–grass mixture fields. Three transfer learning methods, namely, fine-tuned DeepLab V3+, SegNet, and fully convolutional network-8s (FCN-8s), were utilized to detect clover fractions (on an area basis). The detected clover fraction (*CF*_*detected*_), together with auxiliary variables, viz., measured clover height (*H*_*clover*_) and grass height (*H*_*grass*_), were used to build multiple linear regression (MLR) and back propagation neural network (BPNN) models for BC estimation. A total of 347 clover–grass images were used to build the estimation model on clover fraction and BC. Of the 347 samples, 226 images were augmented to 904 images for training, 25 were selected for validation, and the remaining 96 samples were used as an independent dataset for testing. Testing results showed that the intersection-over-union (*IoU*) values based on the DeepLab V3+, SegNet, and FCN-8s were 0.73, 0.57, and 0.60, respectively. The root mean square error (*RMSE*) values for the three transfer learning methods were 8.5, 10.6, and 10.0%. Subsequently, models based on BPNN and MLR were built to estimate BC, by using either *CF*_*detected*_ only or *CF*_*detected*_, grass height, and clover height all together. Results showed that BPNN was generally superior to MLR in terms of estimating BC. The BPNN model only using *CF*_*detected*_ had a *RMSE* of 8.7%. In contrast, the BPNN model using all three variables (*CF*_*detected*_, *H*_*clover*_, and *H*_*grass*_) as inputs had an *RMSE* of 6.6%, implying that DeepLab V3+ together with BPNN can provide good estimation of BC and can offer a promising method for improving forage management.

## Introduction

Forage crops are the main source of nutrition for ruminant animals such as cows. High-quality forages promote the growth of ruminants and result in more efficient production and high-quality animal products. Many grasslands, for either grazing or harvest, include a mixture of grass and clover, or other legumes (Steinshamn and Thuen, 2008). A grass–legume polyculture can use the resources of water, soil nutrients, space, light, and heat more efficiently and can improve the yield and quality of the forage. Legumes generally have a higher protein concentration than grasses, due to their ability to biologically fix nitrogen in symbiosis with rhizobia bacteria. A forage with a high botanical composition (BC) can thus provide a high quality of feed for livestock. The competition between clover and grass is largely impacted by cutting management and N fertilization. Accurate estimation of BC (i.e., the fraction of clover by dry weight, hereinafter referred to as BC) in the mixed clover–grass fields is necessary for fertilization decision making (Nyfeler et al., 2011), estimation of forage quality (Parsons et al., 2013), and general assessment of the performance of grassland.

Traditionally, BC is determined either by hand separation in the laboratory or by visual assessment in the field, which is labor-intensive and inaccurate (Zhou et al., 2019). Alternatively, image analysis methods have been tested for crop species classification, due to species-specific color and/or texture. For instance, Bakhshipour and Jafari (2018) developed artificial neural networks (ANNs) and support vector machine (SVM) classifiers utilizing shape factors, moment invariant features, and Fourier descriptors. ANN and SVM correctly identified from the weeds 93.3 and 96.7%, respectively, sugar beet plants. In the case of artificially sown pastures, where clover and grass were grown separately in clusters, Ahmad et al. (2018) designed edge orientation features and shape matrix histograms as inputs to train AdaBoost and naive Bayes classifiers, which discriminated the clover and grass with accuracy of 98.4%. Methods based on principal component analysis (PCA), Sobel edge extraction, and eroding and dilating operations were also employed for white clover detection (Bonesmo et al., 2004). However, mixed clover and grass in fields, as used in the current study, are far more complicated and represent the real conditions on farms. Most mathematical morphology methods mentioned above had difficulty identifying tiny, dense, and heavily obscured clover fractions (CFs). For typically grown legume–grass mixtures, McRoberts et al. (2016) established an estimation model of grass and clover BC by using local binary patterns (LBPs) extracted from clover–grass images, with a correlation coefficient of 0.895. However, their methods still relied on ergodic feature extraction operating in the LBP algorithm and empirical regression analysis, which could not directly present pixel wise classification information.

Deep learning methods have been widely applied in many fields such as agriculture (Quan et al., 2019), industry (Li et al., 2018), military (Yang Z. et al., 2019), and medicine (Choi, 2018). The advantage of deep learning methods lies in their capacity for extracting deeper object features in a complex scene. There is some existing research that estimates characteristics of crops using deep learning methods. A number of deep learning models were applied to specific recognition tasks where the object’s surface colors were similar to the backgrounds (Koirala et al., 2019). Examples of applications include convolutional neural network (CNN) and Yolo for wheat and barley yield prediction from remote sensing images (Nevavuori et al., 2019), estimation of the number of green apple fruits (Tian et al., 2019), recognition of diseases and pests of tomatoes (Fuentes et al., 2017), and detection of ender tea shoots for picking (Yang H. et al., 2019).

The above-mentioned deep learning methods can acquire the accurate position of objects. However, the semantic segmentation deep learning method can assign specific classification information to each pixel, rather than solely obtaining the position of objects. Sadeghi-Tehran et al. (2019) combined the simple linear iterative clustering (SLIC) algorithm with deep CNN, for semantic segmentation of green, yellow-green, and yellow spikes in a wheat field. Skovsen et al. (2017) used the fully convolutional network-8s (FCN-8s) model to detect clover and grass with a pixel classification accuracy of 83.4%. Kestur et al. (2019) designed a MangoNet network architecture by improving the deep CNN, which was applied to semantic segmentation for the detection of tiny mango fruits on one side of a complete mango tree. Compared with the FCN, the MangoNet method had improved performance. In some cases, rectangular localization generated from the traditional deep learning methods may not suitable for detailed information acquirement of tiny objects with different shapes and mutual obscurement. Therefore, a semantic segmentation network based on deep learning is a potential method for estimation of BC.

Transfer learning can apply generalizable knowledge obtained from one task to another different but related task. Different transfer learning-based classification models such as VGG-19 pre-trained on the ImageNet database and Faster RCNN with ResNet-50 and InceptionV2 pre-trained on the COCO database were used in a recent study for the detection of plant species and diseases (Suh et al., 2018; Selvaraj et al., 2019). Espejo-Garcia et al. (2020) confirmed that the method of combining transfer learning based on the DenseNet pre-trained on ImageNet database with SVM could accomplish weed identification with a promising accuracy of 99.29%. In some cases, the fine-tuning method of using a small number of samples could obtain satisfactory detection results (Pereira et al., 2019). This kind of method utilizes existing network structures in which the weights are initialized on large datasets, thus leading to fast convergence.

The objective of this study was to detect clover among farm-grown clover–grass mixtures by transfer learning-based image analysis. Ultimately, the model detected CF was used either alone or together with other variables to estimate BC.

## Materials and Methods

### Site Description and Image Acquisition

**FIGURE 1:**
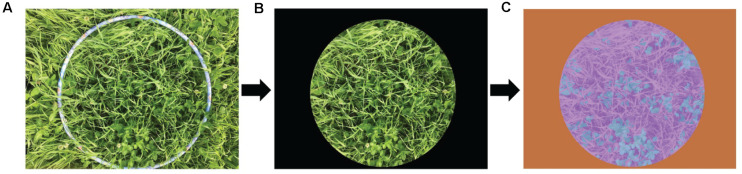
Extraction of the representative sample area and progression through to manual labeling of the image. **(A)** Original image. **(B)** Extracted representative sample area. **(C)** Manually labeled image; blue represents clover, purple represents grass, and orange represents black background.

Three years of image acquisition was carried out at several sites in Northern Sweden: Röbäcksdalen (63°48′ N, 20°14′ E), Ås (63°25′ N, 14°56′ E), Offer (63°14′ N, 17°75′ E), and Öjebyn (65°21′ N, 21°23′ E), from June to August in 2017 and 2018, and from June to July in 2019. Each image was taken from a delineated area using a round hoop (50-cm diameter) (Figure 1A) using an Apple iPhone SE camera with RGB color space, a resolution of 4,032 × 3,024 pixels and JPEG image storage format. In order to reduce the effect of environmental factors, the clover–grass images were all captured in sunny and low wind conditions with stable illumination. A total of 347 images were taken across different growth stages, sites, and years, representative of the growing season in Northern Sweden. All images were taken of mixtures of Timothy (*Phleum pratense* L.) and Red clover (*Trifolium pratense* L.). During each plant sampling, the average heights of red clover and grass were measured with a meter stick.

Shortly after image acquisition, the samples within the round hoop were harvested at a 7-cm stubble height and manually separated into clover and grass fractions, which were dried at 60°C for 48 h in a fan-forced oven until constant weight. Subsequently, BC was defined as the clover dry weight as a percentage of total clover and grass dry weight. The CF was defined as the clover pixel area as a percentage of total clover and grass pixel area. The BC and CF are thus as follows:

(1) $${\mathrm{BC}}_{\mathrm{measured}}=\frac{W_{\mathrm{clover}}}{W_{\mathrm{clover}}+W_{g\,r\,a\,s\,s}}\times 100\%$$

(2) $${\mathrm{CF}}_{\mathrm{detected}}=\frac{D_{\mathrm{clover}}}{D_{\mathrm{clover}}+D_{\mathrm{grass}}}\times 100\%$$

(3) $${\mathrm{CF}}_{\mathrm{measured}}=\frac{M_{\mathrm{clover}}}{M_{\mathrm{clover}}+M_{\mathrm{grass}}}\times 100\%$$

where *BC*_*measured*_ indicates measured BC; *W*_*clover*_ and *W*_*grass*_ are the dry matter (DM) weight of clover and grass, respectively. *CF*_*detected*_ represents the detected CF by the tested transfer learning methods. *D*_*clover*_ and *D*_*grass*_ are the detected pixel-level area of the clover and grass fractions, respectively, obtained by transfer learning methods. *CF*_*measured*_ is the measured CF, and these values are used as ground truth values. *M*_*clover*_ and *M*_*grass*_ are the pixel-level areas of manually labeled clover and grass fractions, respectively.

### Image Preprocessing

The total 347 images were cropped to remove areas outside the edge of the round hoop using Photoshop software (Figure 1B). All cropped images were manually categorized as clover, grass, or black background, using the Image Labeler Toolbox in Matlab R2019a software (Figure 1C). The sample images were hypothetically marked as grass, represented in purple. Due to the relatively simple pixel features of the black background, the flood fill operation was performed to determine the category labels of the black background pixels and further generate an orange mask. There were some dark areas that were misjudged as the black background. After manual detail repairs using pixel label, the sample images were classified into forage and black background. The pixels on the clover contours were artificially depicted from the forage, thus automatically generating the marked clover regions represented by blue.

Image processing was completed on a PC with the following specifications: Windows 10 operating system, 3.60 GHz processor (Intel Core i7-9700K), 64 GB RAM, 2 TB hard disk, and 11 GB GPU (NVIDIA GeForce RTX 2080 Ti). All image processing and analyses were run in Matlab R2019a.

### Transfer Learning-Based Methods for Clover Detection

Three deep learning methods, namely, DeepLab V3+, SegNet, and FCN-8s, were introduced and fine-tuned by collected small forage dataset to detect clover pixels/regions and then to calculate the CF of each image. A total of 251 images acquired in 2017 and 2018 were used for model training and validation. For 226 of the 251 images, three image data augmentation methods including random reflection in the left–right direction and horizontal and vertical translation were carried out to generate a training set (904 images). The remaining 25 images were applied for validation. Images acquired in 2019 (96 images) were used to test the models built from previous steps.

#### Clover Detection Based on the DeepLab V3+ Model

**FIGURE 2:**
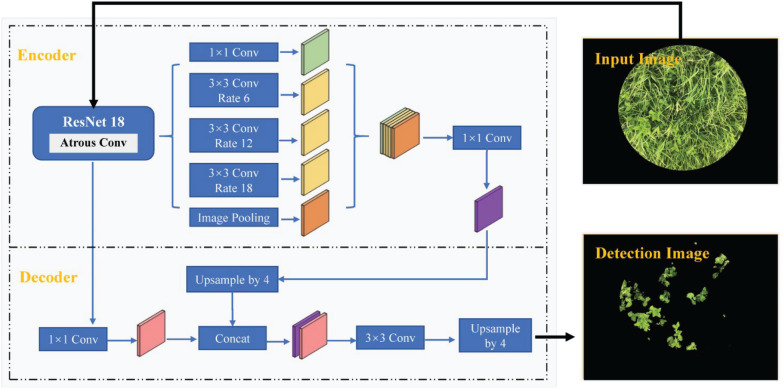
Network structure of the DeepLab V3+ model.

DeepLab V3+ is a deep learning-based semantic segmentation method proposed by Chen et al. (2018), which fuses encoding and decoding structures to accomplish pixel classification and object detection. The network structure of the DeepLab V3+ model used in this study is depicted in Figure 2. An image feature map, obtained from the ResNet-18 network backbone trained on the ImageNet database, was used as input for the atrous spatial pyramid pooling (ASPP) structure including one 1 × 1 and three 3 × 3 convolution layers with atrous rates of 6, 12, and 18. In this manner, multi-scale image features were extracted and pooled to obtain the high-level features. The low-level feature directly extracted from the ResNet-18 network was also processed in the decoder module at the same time. The concat, consisting of the upsampling high-level features and low-level features, was further processed by the convolution and upsampling processing. The network weights were fine-tuned by training a small set of samples images. DeepLab V3+ network ultimately accomplished pixel classification and image segmentation.

In this study, the weights of the DeepLab V3+ network were initiated based on a ResNet-18 pre-trained on the ImageNet database for image classification and later trained with the optimizer of the stochastic gradient descent with momentum (SGDM), initial learn rate of 10^–3^, mini-batch size of 2, weight decay of 5 × 10^–3^, momentum of 0.9, and maximum epochs of 30.

#### Clover Detection Based on the SegNet Model

**FIGURE 3:**
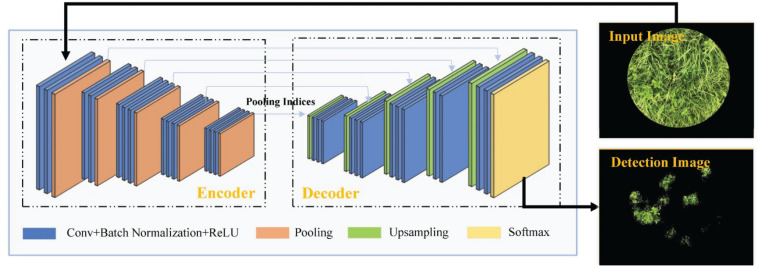
Network structure of the SegNet model.

SegNet is a network model based on the symmetrical encoding and decoding structure designed by Badrinarayanan et al. (2017). This network is a modified version of the VGG-16 model trained on the ImageNet database, as depicted in Figure 3. In the encoder module, the first 13 convolutional layers and five pooling layers of VGG-16 were divided into five encoder blocks and labeled by pooling indices. All encoder blocks had symmetrical decoder blocks. Image features from the encoder were delivered to the decoder through the pooling indices. In this way, the image pixels were classified to accomplish semantic segmentation. Specific network structure is described in Majeed et al. (2020). In this study, the SegNet was initiated by using the VGG-16 weights pre-trained on the ImageNet database, the optimizer of the SGDM, initial learn rate of 10^–3^, mini-batch size of 2, weight decay of 5 × 10^–3^, momentum of 0.9, and maximum epochs of 30 and was eventually optimized using transfer learning based on the small sample fine-tuning method.

#### Clover Detection Based on the Fully Convolutional Network-8s Model

**FIGURE 4:**
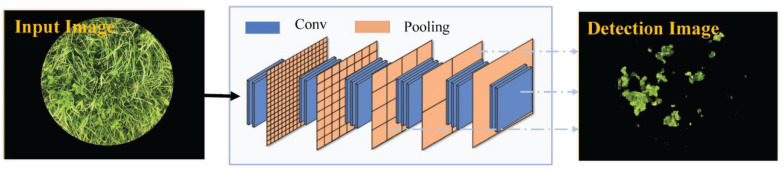
Network structure of the fully convolutional network-8s (FCN-8s) model.

Fully convolutional network-8s is a network framework developed by Shelhamer et al. (2017) that can be applied to image semantic segmentation. The FCN-8s network is depicted in Figure 4. The last fully connected layers in the classic VGG-16 model trained on the ImageNet database were replaced by fully convolutional layers, so as to extract the image features with low resolution. In order to recover the lost spatial information, the segmentation result was further refined by fusing low-level features (Conv 3 and Conv 4). Details of the network structure of FCN-8s are described in Skovsen et al. (2017). In this study, the initialization weights were based on VGG-16 weights pre-trained on the ImageNet database and would be constantly updated in the sample training processing. Adaptive moment estimation (Adam) utilized the first-order and second-order moment estimation of gradient to dynamically adjust the learning rate of each parameter so that the parameters were relatively stable in each iterative learning. We chose Adam as the optimization algorithm and the other training parameters included initial learn rate of 10^–3^, mini-batch size of 2, weight decay of 5 × 10^–3^, momentum of 0.9, and maximum epochs of 30.

### Regression Models for Botanical Composition

It is reasonable to assume that the area-based CF is correlated with weight-based BC. However, BC is not only a function of CF but also a function of other factors such as grass and clover height. To test these relationships, we built regression models to estimate *BC*_*measured*_ either using CF as the only explanatory variable or using three variables (CF, grass height, and clover height). The multiple linear regression (MLR) and back propagation neural network (BPNN) methods were used to build estimation models. We implemented MLR and BPNN methods using the Statistics Toolbox and Neural Network Toolbox in Matlab R2019a software, respectively. These were used to determine whether adding plant height variables could improve the accuracy of BC prediction. The principles of MLR and BPNN are described in Mouazen et al. (2010) and González-Sanchez et al. (2014), respectively.

In this study, the MLR model was established according to Eq. 4.

(4) $$Y=\beta +a_{1}{C\,F}_{d\,e\,t\,e\,c\,t\,e\,d}+a_{2}H_{g\,r\,a\,s\,s}+a_{3}H_{c\,l\,o\,v\,e\,r}$$

where *Y* represents predicted BC; β is a constant; *a*_1_, *a*_2_, and *a*_3_ are regression coefficients; and *H*_*grass*_ and *H*_*clover*_ indicate the average heights of grass and clover within a round hoop, respectively.

**FIGURE 5:**
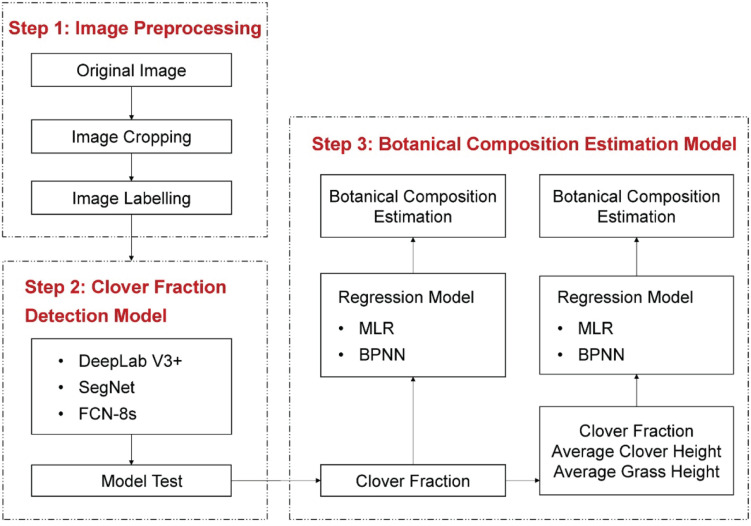
Overall workflow of the current study on clover detection and botanical composition estimation.

In the BPNN model, the network could be divided into input layer, hidden layer, and output layer. The number of neurons in the hidden layer was set to 5. The BPNN model used the Levenberg–Marquardt method for optimization of weight and bias parameters and was trained using maximum epochs of 10^3^, learning rate of 0.6, and goal error of 10^–5^. The overall estimation process of BC is depicted in Figure 5. A total of 347 clover–grass sample images were divided into a training set (251 samples) and a testing set (96 samples).

### Evaluation Criteria

In order to evaluate the performances of the proposed methods for CF and BC estimation, *Accuracy*, intersection-over-union (*IoU*), root mean square error (*RMSE*), *R*^2^, regression line slope *b*, and intercept *a* were calculated. *Accuracy*, *IoU*, *RMSE*, and *R*^2^ indices were calculated by Eqs 5–8.

(5) $$\mathrm{Accuracy}=\frac{\mathrm{TP}}{\mathrm{TP}+\mathrm{FN}}$$

(6) $$\mathrm{IoU}=\frac{\mathrm{TP}}{\mathrm{TP}+\mathrm{FN}+\mathrm{FP}}$$

(7) $$\mathrm{RMSE}=\sqrt{\frac{1}{m}\times \sum_{i=1}^{m}{\left(P_{i}-O_{i}\right)}^{2}}$$

(8) $$R^{2}=1-\frac{\sum_{i=1}^{m}{\left(P_{i}-O_{i}\right)}^{2}}{\sum_{i=1}^{m}{\left(\bar{O}-O_{i}\right)}^{2}}$$

where true positive (*TP*) is the number of correctly predicted clover pixels by transfer learning methods, false negative (*FN*) indicates the number of pixels that actually belong to clover area but are misjudged as grass pixels, false positive (*FP*) represents the number of pixels that actually belong to grass area but are misjudged as clover pixels, *P*_*i*_ and *O*_*i*_ are the *i*-th predicted and observed values in sample data, *m* is the number of samples, and $\bar{O}$ is the average value of observed data.

## Results

### Detection Performance of Clover Fraction Based on DeepLab V3+, SegNet, and Fully Convolutional Network-8s Models

During the training process, the variation in accuracy and loss with the increase of iteration time is depicted in Supplementary Figure 1. The accuracy and loss gradually converged after increasing and decreasing, respectively. And the detection results of the testing set based on the three transfer learning models are shown in Table 1. DeepLab V3+ had the best performance with the highest *Accuracy* of 0.95, which was 0.13 higher than SegNet and 0.09 higher than FCN-8s. The *IoU* of DeepLab V3+ was 0.73, which was 0.16 higher than SegNet and 0.13 higher than FCN-8s.

**TABLE 1:** Performance of DeepLab V3+, SegNet, and FCN-8s methods for clover fraction detection of the testing test.

**Labels**	***n*_*p*_**	***Accuracy***			***IoU***		
		**DeepLab V3+**	**SegNet**	**FCN-8s**	**DeepLab V3+**	**SegNet**	**FCN-8s**
Clover	1.01E+08	0.95	0.82	0.86	0.73	0.57	0.60
Grass	3.98E+08	0.93	0.87	0.89	0.91	0.81	0.86
Black background	6.71E+08	1.00	0.98	1.00	1.00	0.97	1.00

*n_*p*_, the total number of tested pixels; *Accuracy*, the classification accuracy of pixels; *IoU*, the intersection over union; FCN-8s, fully convolutional network-8s.*

**FIGURE 6:**
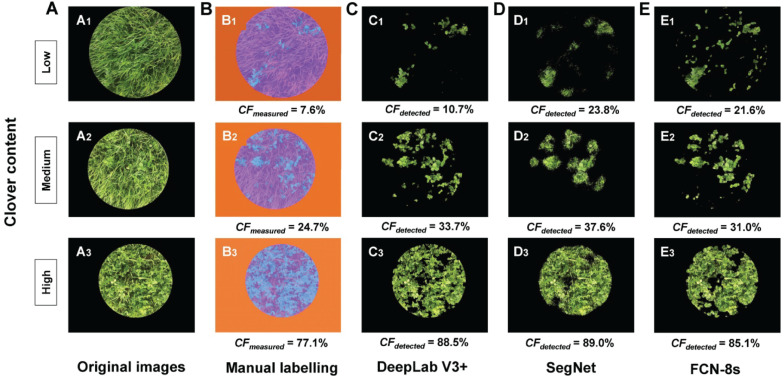
Comparison of clover fraction detection results between different transfer learning methods. **(A)** Original images. **(B)** Manual labeling methods, used as the reference: blue represents clover, purple represents grass, and orange represents black background. **(C)** Clover detected by the DeepLab V3+ method. **(D)** Clover detected by the SegNet method. **(E)** Clover detected by the FCN-8s method. *CF*_*measured*_ represents the measured clover fraction obtained by manual labeling (%), and *CF*_*detected*_ represents the detected clover fraction obtained by transfer learning methods (%).

Three examples (representing low, middle, and high CF) of *CF*_*detected*_ are presented in Figure 6 to intuitively compare the different transfer learning-based methods. DeepLab V3+ can be effectively trained to detect the clover even under conditions of serious mutual obscurement between clover and grass (Figure 6C). CFs in Figure 6D, obtained from the SegNet method, showed noisy effects along the clover boundary. The detection performance of the FCN-8s method was in between (Figure 6E1). However, with a high clover content in the sample images, the detected CFs were closer to the true CFs by using the FCN-8s network (Figures 6E2,E3). Comparing the three transfer learning methods, DeepLab V3+ was overall more effective for estimating *CF*_*measured*_.

**FIGURE 7:**
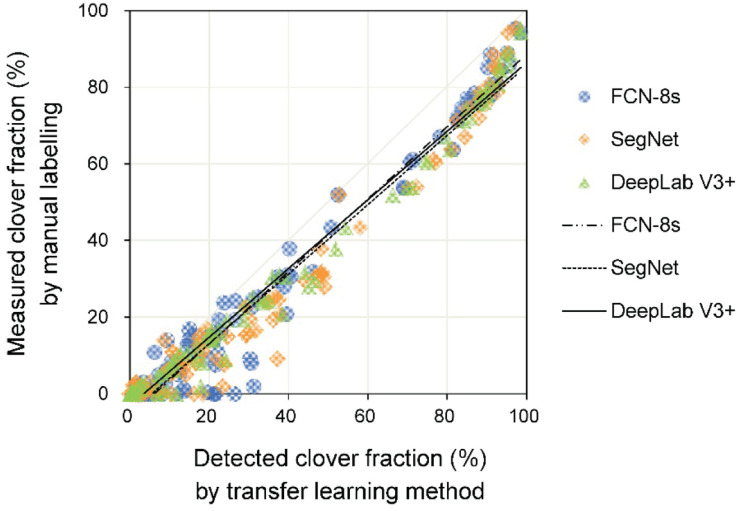
The relationship between measured and detected clover fraction by three transfer learning models.

The linear relationships between measured and detected CFs for the three transfer learning methods are shown in Figure 7 and Table 2. The results showed that the *R*^2^ values for the different models were all above 0.96, implying satisfactory performance of different models for CF estimation. The DeepLab V3+ based model had the lowest *RMSE* (8.5%), while SegNet and FCN-8s *RMSE*s were 10.6 and 10.0%, respectively. A few data points are notable along the horizontal axis, indicating misclassification of clover. The fuzzy boundaries between clover and grass seriously influenced the detection effects of the CF. Particularly for the SegNet and FCN-8s, there were numerous instances at low levels of *CF*_*measured*_, where the *CF*_*detected*_ was much higher.

**TABLE 2:** Estimation result statistics of clover fractions from clover–grass mixtures with different height differences by three deep learning methods.

**Groups**	***n*_*s*_**	***R*^2^**	***RMSE* (% CF)**	***b***	***Prob. b* = 1**	***a***	***Prob. a* = 0**
**Pooled data**							
DeepLab V3+	96	0.98	8.5	0.89	<0.001	−3.07	<0.001
SegNet	96	0.96	10.6	0.90	<0.001	−4.94	<0.001
FCN-8s	96	0.96	10.0	0.94	<0.001	−5.80	<0.001
**Grass higher than clover (*H*_*grass*_−*H*_*clover*_ > 10 cm)**							
DeepLab V3+	46	0.95	7.6	0.71	<0.001	−1.31	0.021
SegNet	46	0.88	9.8	0.68	<0.001	−2.32	0.009
FCN-8s	46	0.78	11.3	0.71	<0.001	−4.00	0.003
**Similar height (0 cm ≤ *H*_*grass*_−*H*_*clover*_ ≤ 10 cm)**							
DeepLab V3+	41	0.99	9.5	0.88	<0.001	−2.92	<0.001
SegNet	41	0.97	11.9	0.89	<0.001	−4.97	0.003
FCN-8s	41	0.98	8.5	0.90	<0.001	−2.24	0.005
**Clover higher than grass (*H*_*grass*_−*H*_*clover*_ < 0 cm)**							
DeepLab V3+	9	0.99	7.9	0.98	<0.001	−5.92	0.174
SegNet	9	0.97	8.1	1.01	<0.001	−7.86	0.220
FCN-8s	9	0.98	8.2	1.06	<0.001	−12.78	0.048

*CF, clover fraction; *n*_*s*_, the number of samples; *RMSE*, root mean square error; *b*, slope; *Prob*, probability value; *a*, intercept; FCN-8s, fully convolutional network-8s.*

### Estimating Clover Fraction for Different Clover–Grass Growing Heights

**FIGURE 8:**
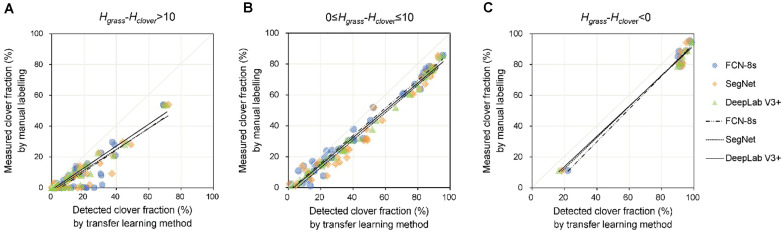
Comparison of transfer learning models [DeepLab V3+, SegNet, and fully convolutional network-8s (FCN-8s)] used to estimate the clover fraction, sorted by different relationships between clover and grass height. **(A)** *H*_*grass*_ – *H*_*clover*_ > 10 cm. **(B)** 0 cm ≤ *H*_*grass*_ – *H*_*clover*_ ≤ 10 cm. **(C)** *H*_*grass*_ – *H*_*clover*_ < 0 cm. *H*_*grass*_ represents the average grass height (cm) in a mixed clover–grass sample sward, and *H*_*clover*_ represents the average clover height (cm).

In addition to the forage varieties and different illumination conditions, the sward height and distribution of species within the canopy may impose confounding effects on the relationship between the transfer learning detected and human operator measured pixel-level areas of CFs. In view of these factors, we focused on the impact of clover–grass height difference (*H*_*grass*_ − *H*_*clover*_) on the performance of different transfer learning models (Figure 8 and Table 2). When the height difference was over 10 cm,

the slope *b* values were significantly less than one, and intercept *a* values were closer to zero. At low values of CF, there was little bias; however, as CF increased, the estimated CF became more biased (Figure 8A). Among the three transfer learning-based methods, DeepLab V3+ presented the best performance for CF estimation (*R*^2^ = 0.95, *RMSE* = 7.6%, slope *b* = 0.71, and intercept *a* = −1.31). For height difference categories of 0 < *H*_*grass*_ − *H*_*clover*_ ≤ 10 cm and *H*_*grass*_ − *H*_*clover*_ < 0 cm, the *R*^2^ values of DeepLab V3+, SegNet, and FCN-8s were 0.99, 0.97, and 0.98, respectively. For the group 0 < *H*_*grass*_ − *H*_*clover*_ ≤ 10 cm, *RMSEs* of DeepLab V3+, SegNet, and FCN-8s models were 9.5, 11.9, and 8.5%, respectively. For the group *H*_*grass*_ − *H*_*clover*_ < 0, i.e., clover higher than grass, *RMSE*s for the CF estimation model were lower than those of the other groups (7.9, 8.1, and 8.2 for DeepLab V3+, SegNet, and FCN-8s, respectively). As the height difference decreased, the slope *b* was closer to one.

### Estimation of Botanical Composition

**FIGURE 9:**
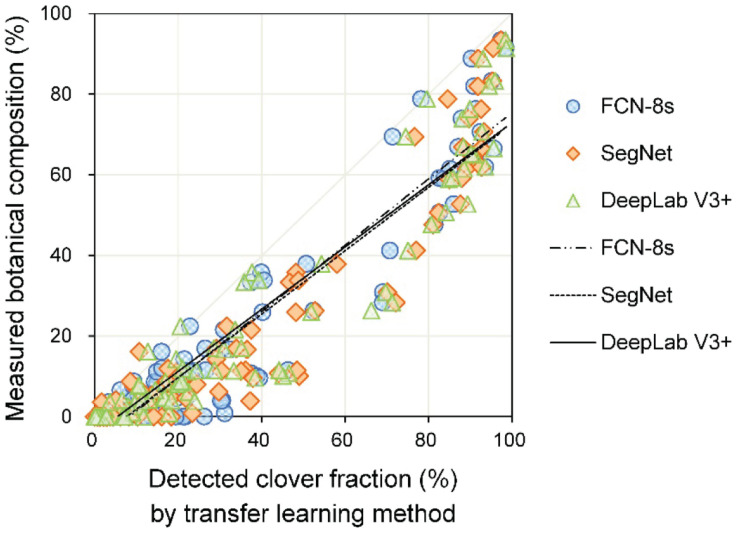
Linear regression of the detected clover fraction and measured botanical composition.

The linear regression between the estimated CF (*CF*_*detected*_) and the measured BC (*BC*_*measured*_) is plotted in Figure 9. For the pooled dataset, the *R*^2^ values were all approximately 0.90, and the *RMSE* values were approximately 17% (Table 3).

**TABLE 3:** Linear regression result statistics of the detected clover fraction and measured botanical composition.

**Methods**	***n*_*s*_**	***R*^2^**	***RMSE* (% BC)**	***b***	***Prob. b* = 1**	***a***	***Prob. a* = 0**
DeepLab V3+	96	0.90	16.8	0.77	<0.001	−4.22	0.001
SegNet	96	0.89	17.8	0.79	<0.001	−6.05	<0.001
FCN-8s	96	0.90	16.8	0.82	<0.001	−6.90	<0.001

*BC, botanical composition; *n*_*s*_, the number of samples; *RMSE*, root mean square error; *b*, slope; *Prob*, probability value; *a*, intercept; *CF*_*detected*_, detected clover fraction; *H*_*clover*_, average height of clover in sample; *H*_*grass*_, average height of grass in sample; FCN-8s, fully convolutional network-8s.*

**FIGURE 10:**
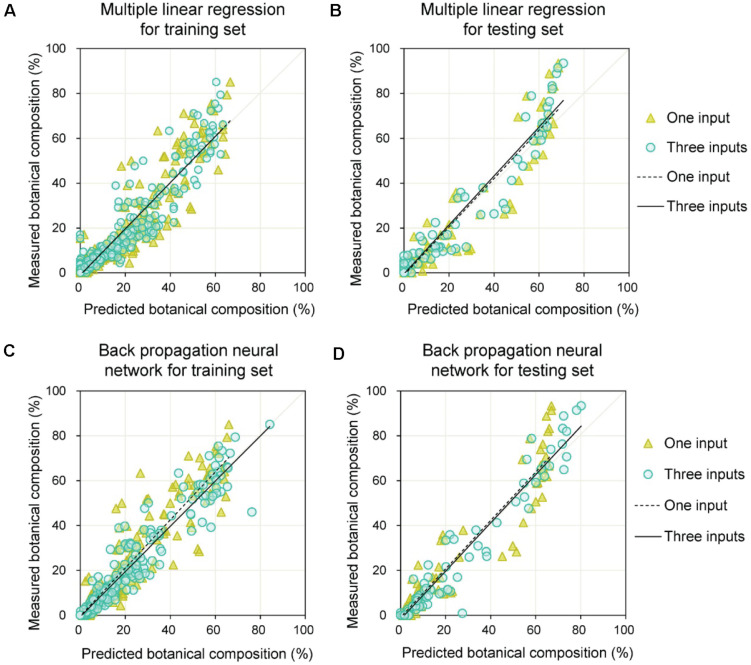
Prediction of botanical composition for training and testing sets by combining DeepLab V3+ model with multiple linear regression or back propagation neural network methods. **(A)** Training set results obtained by multiple linear regression. **(B)** Testing set results obtained by multiple linear regression. **(C)** Training set results obtained by back propagation neural network. **(D)** Testing set results obtained by back propagation neural network.

Due to the potential effect of the relative height of clover and grass, grass height and clover height were added as auxiliary variables to improve the estimation of BC. The models using MLR and BPNN for BC estimation are shown in Figure 10. From Table 4, the *b* values were approximately one, and the *a* values varied between minus two and zero. Compared with models that only used *CF*_*detected*_, the three-input models significantly improved the estimation of BC (in terms of *R*^2^, *RMSE*, *b*, and *a* of training and testing sets). For the testing set, the *RMSE*s with three inputs were 6.6 and 7.5% for the BPNN and MLR, respectively, which were lower than those of BPNN and MLR with one input (*RMSE* = 8.7%).

**TABLE 4:** Estimation result statistics of botanical composition for training and testing sets by combining DeepLab V3+ model with multiple linear regression or back propagation neural network methods.

**Explanatory variables**	***n*_*s*_**	***R*^2^**	***RMSE* (% BC)**	***b***	***Prob. b* = 1**	***a***	***Prob. a* = 0**
**Multiple linear regression for training dataset**							
*CF*_*detected*_, *H*_*clover*_, *H*_*grass*_	251	0.88	7.2	1.03	<0.001	−0.94	0.154
*CF*_*detected*_	251	0.84	8.1	1.03	<0.001	−1.14	0.131
**Multiple linear regression for testing dataset**							
*CF*_*detected*_, *H*_*clover*_, *H*_*grass*_	96	0.94	7.5	1.09	<0.001	−0.46	0.624
*CF*_*detected*_	96	0.91	8.7	1.08	<0.001	−1.08	0.354
**Back propagation neural network for training dataset**							
*CF*_*detected*_, *H*_*clover*_, *H*_*grass*_	251	0.92	5.9	1.01	<0.001	−0.71	0.188
*CF*_*detected*_	251	0.86	7.7	1.07	<0.001	−0.31	0.657
**Back propagation neural network for testing dataset**							
*CF*_*detected*_, *H*_*clover*_, *H*_*grass*_	96	0.95	6.6	1.07	<0.001	−1.82	0.039
*CF*_*detected*_	96	0.91	8.7	1.08	<0.001	−0.87	0.451

*BC, botanical composition; *n*_*s*_, the number of samples; *RMSE*, root mean square error; *b*, slope; *Prob*, probability value; *a*, intercept; *CF*_*detected*_, detected clover fraction; *H*_*clover*_, average height of clover in sample; *H*_*grass*_, average height of grass in sample.*

## Discussion

A convenient smart phone camera was used to capture the mixed clover–grass images to estimate CF and BC by image analysis. All proposed transfer learning-based models could satisfactorily detect clover regions within images. SegNet and FCN-8s models had the same encoding structure, which took advantage of different decoding layers. The SegNet model maintained the integrity of high-frequency contents through index transmission from all encoder pooling layers to the decoder part, whereas it also ignored information from nearby pixels. The performance of SegNet network presented obscure detection results as a whole, together with some noise points scattered around the clover areas (Figure 6D). Compared with the SegNet network, FCN-8s only employed low-level features from Conv 3 and Conv 4 for the decoder module. However, FCN-8s revealed greater robustness for detecting clover pixels than SegNet. These results might be derived from the fact that the encoder transmitted too many high-resolution features to the decoder in the SegNet model, leading to feature information redundancy. Compared with SegNet and FCN-8s, DeepLab V3+ had the highest *Accuracy* and *IoU*. This was mainly attributed to the ResNet-18 backbone that the DeepLab V3+ network used, whereas SegNet and FCN networks were all based on a VGG-16 backbone. Compared with the VGG-16, ResNet-18 has a light network backbone with less computation (He et al., 2016). Combining with the superiority of atrous convolution that effectively controlled image feature resolution from the ResNet-18 backbone containing the residual module, DeepLab V3+ could better estimate effects of the CF.

In most cases, CF was overestimated by the three transfer learning models (Figure 7). This likely stemmed from the misjudgment of some grass pixels. Due to extremely similar color features between clover and grass, some grass leaves that were extending to cover parts of the clovers and similar connected domains represented by grasses could not be detected accurately. This situation resulted in an excessively high *D*_*clover*_, thus generating some *CF*_*detected*_ values that were significantly higher than *CF*_*measured*_ values. Overall, the increased height difference between grass and clover (*H*_*grass*_ − *H*_*clover*_) caused lower performance of the CF estimation model (Figure 8 and Table 2). This was because the height difference between grass and clover led to mutual obscurement of the two species. When the grass height increased relative to clover, more clover was obscured by the grass, and more shadows appeared in the sample image. This caused more clover pixels to be undetected and shadowed regions to be misjudged by image analysis. The statistical results for DeepLab V3+ were slightly better than for SegNet and FCN-8s. When the height difference (*H*_*grass*_ − *H*_*clover*_) was between 0 and 10 cm, the slopes were closer to one, and the models were less biased (Figure 8B). Although the values for slope and intercept were similar between models, the *R*^2^ values were higher and *RMSE* values were lower for DeepLab V3+.

Machine learning-based image analysis has been employed for crop species classification; e.g., clover, grass, weed, and vegetable in some studies, for instance, Bonesmo et al. (2004) and Himstedt et al. (2012) confirmed the feasibility of dilation and erosion methods for clover and grass fraction estimation. However, the crop growing conditions were not under natural field conditions, unlike with this study. Few studies have incorporated deep learning methods into forage BC detection, especially using transfer learning-based semantic segmentation. Abdalla et al. (2019) proved that the transfer learning method based on the VGG-16 network pre-trained on the ImageNet database could achieve semantic segmentation of oilseed rape images from a field with high weed pressure. Our results corroborated those of Abdalla et al. (2019), which showed that transfer learning has great potential to estimate plant coverage ratio in extremely complex growing conditions with variable illumination.

The relationship between *CF*_*detected*_ and *BC*_*measured*_ was strong but nonlinear (Figure 9 and Table 3). Although the weight-based BC was correlated with the area-based CF, there were other factors that influenced it. Models using *CF*_*detected*_, *H*_*clover*_, and *H*_*grass*_ improved the BC estimation compared with only using *CF*_*detected*_ (Figure 10 and Table 4). This was mainly because species-specific DM is a function of both species-specific canopy coverage and corresponding plant height. Introducing height factors, the proposed method presented satisfactory prediction effects for BC. The results were better with the BPNN method, compared with MLR. For both methods, the statistical results were similar for the training and testing sets, indicating the robustness of the models. Skovsen et al. (2017) verified the BC estimation effects from mixed clover–grass images by utilizing the simulated images to fine-tune FCN-8s network and found that the model could not perfectly predict BC values at moderate levels of clover due to severe obscurement. The results for BC estimation in our study, obtained using DeepLab V3+ and BPNN, offer alternative approaches. Our proposed method provides a reasonable estimation accuracy of BC and was done using simple technology, by training a series of RGB images captured by a camera phone. This confirmed that it was feasible to use transfer learning-based object detection combined with a machine learning-based estimation model for BC prediction under low color contrast, mutual obscurement, and random illumination conditions.

Botanical composition, together with other crop variables (e.g., crop height), can be used to build quantitative models to predict forage quality variables in real time in the field. For example, Parsons et al. (2005) developed field-based tools to help producers to decide the timing of harvest of mixed alfalfa-grass forages. BC is one of the most important characteristics of forages that can inform management. Accurate estimation of BC can assist in harvesting decisions; fertilization decision making, either at the field level or variable rate N fertilization by applying less N to zones of high BC; and providing information to help producers decide when to re-sow forage fields. Our study presented a convenient, nondestructive, and reliable solution for BC estimation by using a camera phone that could be developed into a farmer-useable tool.

One limitation of the proposed method is that serious obscurement and boundary blur greatly influenced the CF detection. Therefore, in future research, an image restoration technology or a deeper semantic segmentation network could be designed to improve the estimation model for BC. The clover contour information may be recovered by utilizing image restoration technologies. Richer image features gained from other advanced semantic segmentation networks can be extracted and selected to accomplish better tiny object detection, so as to obtain more accuracy in estimated BC by improving the estimation of *CF*_*detected*_.

## Conclusion

This study introduced and compared the three transfer learning-based semantic segmentation methods, namely, DeepLab V3+, SegNet, and FCN-8s. The three transfer learning methods showed significant promise for mixed clover–grass images with the RGB color space. In terms of clover detection from an image processing perspective, DeepLab V3+ presented more accurate pixel-level detection results (*Accuracy* of 0.95 and *IoU* of 0.73) than the SegNet and FCN-8s methods. The BC prediction model based on the BPNN was designed by utilizing either only *CF*_*detected*_ or *CF*_*detected*_, *H*_*clover*_, and *H*_*grass*_. Prediction models based on three explanatory variables were significantly superior to the models using only *CF*_*detected*_. The accurate estimation of BC can be used for forage quality evaluation and decision support making regarding fertilizer rates. This could potentially help to optimize N fertilization and reduce the negative effects of excessive N input. The proposed BC estimation model was shown to be valid across different growth stages, years, and sites, implying its robustness for practical application. It is reasonable to assume that the methods proposed in this study could be developed into a real-time monitoring system for farmers.

## Data Availability Statement

The raw data supporting the conclusions of this article will be made available by the authors, without undue reservation.

## Author Contributions

SS, NL, and ZZh conceived and designed the experiments. SS, NL, ZW, and ZZh performed data collection and processing. SS, ZZu, DP, JM, and HF analyzed the data. SS drafted the manuscript. NL, JS, LL, and LZ discussed the analysis results and optimized the experiments. ZZu, DP, JM, JS, YH, and ZZh revised the manuscript. All authors contributed to this manuscript and approved the final version.

## Conflict of Interest

The authors declare that the research was conducted in the absence of any commercial or financial relationships that could be construed as a potential conflict of interest.
