# Supplementary material for: Estimation of Botanical Composition in Mixed Clover–Grass Fields Using Machine Learning-Based Image Analysis
Source: Front Plant Sci. 2021 Feb 11;12:622429. doi: 10.3389/fpls.2021.622429 (PMC7905353; doi:10.3389/fpls.2021.622429)
Supplement: Supplementary file 1 [file Image_1.PDF]

Supplementary Material

Supplementary Material

1 Supplementary Figures

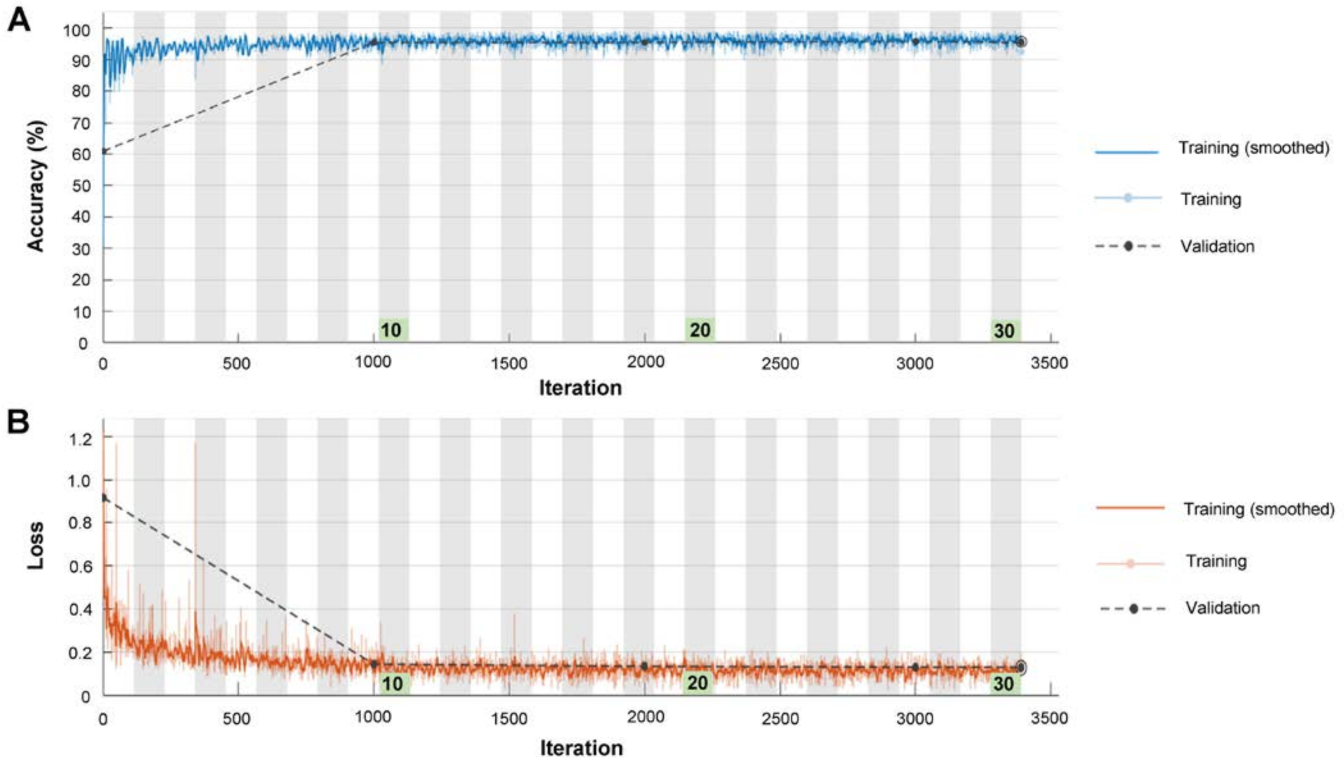

Supplementary Figure 1. The accuracy and loss curves for the training process. (**A**) Accuracy curve. (**B**) Loss curve. 10, 20, and 30 marked in green rectangle blocks represent epochs.
